# Identification and characterization of a *Babesia bigemina* thrombospondin-related superfamily member, TRAP-1: a novel antigen containing neutralizing epitopes involved in merozoite invasion

**DOI:** 10.1186/s13071-020-04469-5

**Published:** 2020-12-01

**Authors:** Valeria Noely Montenegro, Martina Soledad Paoletta, José M. Jaramillo Ortiz, Carlos E. Suarez, Silvina Elizabeth Wilkowsky

**Affiliations:** 1 Instituto de Agrobiotecnología y Biología Molecular (IABIMO) (INTA–CONICET), De Los Reseros y Dr. Nicolás Repetto s/N, P.O. Box 25, B1712WAA Castelar, Buenos Aires Argentina; 2 Department of Veterinary Microbiology and Pathology, Washington State University, Pullman, WA 99164 USA; 3 Animal Disease Research Unit, U.S. Department of Agriculture-Agricultural Research Service (USDA-ARS), Washington State University, 3003 ADBF, P.O. Box 646630, Pullman, WA 99164 USA

**Keywords:** *Babesia bigemina*, Thrombospondin-related anonymous protein 1, TRAP and TRP protein families, Babesiosis

## Abstract

**Background:**

Thrombospondin-related anonymous protein (TRAP) has been described as a potential vaccine candidate for several diseases caused by apicomplexan parasites. However, this protein and members of this family have not yet been characterized in *Babesia bigemina*, one of the most prevalent species causing bovine babesiosis.

**Methods:**

The 3186-bp *Babesia bigemina TRAP*-*1* (*BbiTRAP*-*1*) gene was identified by a bioinformatics search using the *B. bovis TRAP*-*1* sequence. Members of the *TRAP* and TRAP-related protein families (TRP) were identified in *Babesia* and *Theileria* through a search of the TSP-1 adhesive domain, which is the hallmark motif in both proteins. Structural modeling and phylogenetic analysis were performed with the identified TRAP proteins. A truncated recombinant BbiTRAP-1 that migrates at approximately 107 kDa and specific antisera were produced and used in Western blot analysis and indirect fluorescent antibody tests (IFAT). B-cell epitopes with neutralizing activity in BbiTRAP-1 were defined by enzyme-linked immunosorbent assays (ELISA) and invasion assays.

**Results:**

Three members of the TRAP family of proteins were identified in *B. bigemina* (BbiTRAP-1 to -3). All are type 1 transmembrane proteins containing the von Willebrand factor A (vWFA), thrombospondin type 1 (TSP-1), and cytoplasmic C-terminus domains, as well as transmembrane regions. The BbiTRAP-1 predicted structure also contains a metal ion-dependent adhesion site for interaction with the host cell. The TRP family in *Babesia* and *Theileria* species contains the canonical TSP-1 domain but lacks the vWFA domain and together with TRAP define a novel gene superfamily. A variable number of tandem repeat units are present in BbiTRAP-1 and could be used for strain genotyping. Western blot and IFAT analysis confirmed the expression of BbiTRAP-1 by blood-stage parasites. Partial recognition by a panel of sera from *B. bigemina*-infected cattle in ELISAs using truncated BbiTRAP-1 suggests that this protein is not an immunodominant antigen. Additionally, bovine anti-recombinant BbiTRAP-1 antibodies were found to be capable of neutralizing merozoite invasion in vitro.

**Conclusions:**

We have identified the *TRAP* and *TRP* gene families in several *Babesia* and *Theileria* species and characterized BbiTRAP-1 as a novel antigen of *B. bigemina*. The functional relevance and presence of neutralization-sensitive B-cell epitopes suggest that BbiTRAP-1 could be included in tests for future vaccine candidates against *B. bigemina*.

**Graphical Abstract:**

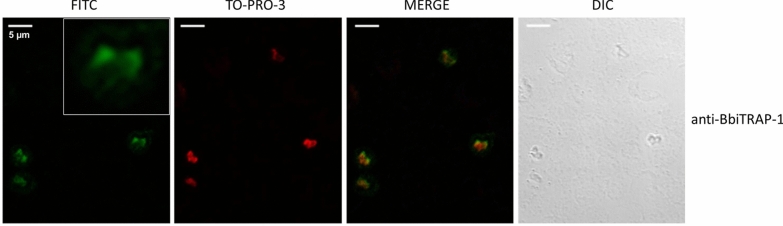

## Background

Parasites of the genus *Babesia* are tick-borne pathogens of human and veterinary importance. In cattle, bovine babesiosis caused by *B. bovis* and *B. bigemina* pose a major constraint to livestock production in tropical and subtropical regions in terms of considerable economic losses [1]. A recent meta-analysis of samples from 62 countries representing six continents revealed a pooled global prevalence of bovine babesiosis of 29% [2].

*Babesia bovis* is the most virulent species in this genus and can occasionally cause neurological manifestations. However, animals infected with *B. bigemina* may also develop severe symptoms of babesiosis, such as high fever, lethargy, anemia, and hemoglobinuria [1]. Of both parasites, *B. bigemina* has also the highest prevalence [2].

Animals that survive primary infections become persistently infected for life and are reservoirs for tick transmission [1]. In the vertebrate host, *Babesia* parasites are obligate intracellular pathogens that exclusively reside inside the erythrocytes [1]. Currently, the use of acaricides and live attenuated vaccines are the only preventive measures used to control outbreaks [3]. Therefore, studies that aim to decipher the process of erythrocyte invasion by *Babesia* infective stages are crucial to develop novel strategies to control the establishment of infection.

*Babesia* parasites belong to the phylum *Apicomplexa*, which is characterized by the presence of a complex of specialized secretory organelles that include rhoptries, micronemes, and spherical bodies. These organelles contain and secrete key proteins implicated in the invasion, establishment, and egress from host cells [4]. In general, the mechanism by which *Babesia* merozoites invade red blood cells is similar to that of other apicomplexan parasites, such as *Plasmodium* and *Toxoplasma*. The process involves attachment to the host cell, reorientation, membrane invagination, and final internalization of the parasite [4]. The initial steps of invasion involve an active specific locomotion movement known as “gliding,” a movement which involves the parasite’s actin–myosin motor [5]. This motor is connected to host-cell receptors through transmembrane proteins that belong to the thrombospondin-related anonymous protein (TRAP) family of proteins [5]. TRAP-1 is a type-1 transmembrane protein that carries two adhesive domains in its extracellular region, a motif similar to the type-1 repeat of thrombospondin (TSP-1) and an A-domain of the von Willebrand factor (vWFA) [6]. Even though the overall mechanism of the parasite’s actin–myosin motor is widely conserved among *Apicomplexa*, differential expression of distinct and species-specific TRAP proteins has been identified among this phylum [7]. However, the exact role played by TRAP-1 and their ligands during the process of invasion in apicomplexan parasites remains undefined.

In 2004, a new *B. bovis* merozoite protein with a domain structure resembling the arrangement of TRAP from *Plasmodium* sporozoites was identified and characterized [6]. This protein was found to be directly involved in both the recognition and invasion processes of bovine erythrocytes. Other TRAPs have also been recently reported in *Babesia orientalis* and *B. gibsoni* [8–10].

The availability of several *Babesia* genomes has facilitated the identification of genes and gene families that are conserved across the phylum and whose presence and function have not been identified in less studied members.

 In this study, we identified members of the *TRAP* gene family in *B. bigemina* and performed a detailed analysis at the genomic and sequence levels. We also searched for distantly TRAP-related proteins and found a new family of thrombospondin-related proteins (TRP) that share some of the structural domains of TRAP proteins. Finally, we analyzed the expression and functional relevance of TRAP-1 in *B. bigemina* merozoites and also investigated the role of this protein as a neutralization-sensitive antigen with vaccine potential.

## Materials and methods

### Identification and characterization of* B. bigemina* TRAP-1

For the identification of the *B. bigemina TRAP*-*1* (*BbiTRAP*-*1*) coding sequence, we performed a TBLASTN search (https://blast.ncbi.nlm.nih.gov/Blast.cgi) in the genome of *B. bigemina* (BOND strain). The search was conducted using the predicted amino acid sequence of the annotated *B. bovis TRAP*-*1* gene as a query (GenBank accession number: EDO06220.1). For in silico topology prediction, concatenated amino acid sequences of the vWFA and TSP-1 domains of BbiTRAP proteins were submitted to the Swiss-model server (http://swissmodel.expasy.org) and three-dimensional (3D) models were constructed. The crystal structure of a fragment containing the vWFA and TSP-1 domains from *Plasmodium vivax* sporozoite surface protein 2 (PDB code: 4hql.2.A, residues 25-283) [11] was used for homology modeling since it was the structure with the highest GMQE score. This score is a quality estimator with values of between 0 and 1 and combines properties from the target–template alignment and the template search method.

The *BbiTRAP*-*1* coding sequence of the BOND strain was used to identify the corresponding genes from the other *B. bigemina* genomes (JG29, S3P, and Puerto Rico [PR] strains) available from the Wellcome Trust Sanger Institute FTP site (ftp://sanger.ac.uk/pub/pathogens/Babesia/) [12]. These *BbiTRAP*-*1* sequences were then translated in silico to confirm the presence of the respective open reading frames. All predicted amino acid sequences were further aligned using the Clustal Ω algorithm (www.ebi.ac.uk/Tools/msa/clustalo/). Percentages of identity and similarity between sequence pairs were calculated using the Sequence Manipulation Suite “Ident and Sim” resource (https://www.bioinformatics.org/sms2/ident_sim.html).

### Identification and characterization of members of the TRAP and TRP family in other species

For the identification the TRAP family in species of the *Babesia* and *Theileria* genera, we performed a database search in PiroplasmaDB (https://piroplasmadb.org/) with the aim to identify all proteins containing the TSP-1 domain (IPR000884) or vWFA domain (IPR002035) [13]. The presence of a signal peptide, transmembrane domains, and other functional domains was recorded. In addition, the occurrence of an acidic cytoplasmatic tail domain (CTD) was analyzed by predicting the isoelectric point using http://isoelectric.org/. The conservation of a tryptophan residue near the C-terminal end of the protein was also analyzed. To determine that a TSP-1-containing protein actually belongs to the TRAP family, the presence of the CTD with the conserved tryptophan residue was considered to be essential [14].

For all TSP-1-containing proteins, the group of orthology to which they belong was determined using the OrthoMCL database (https://orthomcl.org/orthomcl/, version 6.1). Synteny of the chromosomal region containing each gene was evaluated in PiroplasmaDB by visualizing the alignment of the genomic region encompassing each gene in different *Babesia* species. The conservation and distribution pattern of genes surrounding the target locus were also analyzed.

### Phylogenetic analysis

Sequence alignment of the complete *TRAP* gene family from species of the *Babesia* and *Theileria* genera was performed using the MEGA version X tool [15]. Phylogenetic analysis was performed with the maximum likelihood and neighbor joining methods, and tree topologies were compared for a robust phylogeny. In both cases, TRAP of *Plasmodium falciparum* (Access number: PF13_0201) was selected as an outgroup, and bootstrap values were calculated with 1000 pseudo replicates.

### *B. bigemina* strains and genomic DNA isolation

The following Argentinean *B. bigemina* strains were used: S1A, S2A, and M1A (all attenuated vaccine strains) and the pathogenic B38, S3P, and S2P strains. Details of the geographic origin of these strains and the respective multilocus genotypes have been described in a previous publication [16]. For the in silico analysis, the *B. bigemina* genomes of BOND, JG29, and the PR strains were used [12]. We also included sequences obtained from samples of genomic DNA from Brazil, Mexico, and Nayarit State of western Mexico (the latter two kindly provided by Juan Mosqueda, Universidad Autónoma de Querétaro, Mexico).

Extraction of genomic DNA from blood of experimentally inoculated bovines or erythrocyte cultures were performed in phosphate buffered saline (PBS)-washed packed red blood cells by standard phenol–chloroform extraction and ethanol precipitation [17]. DNA was quantified using a NanoDrop 1000 spectrophotometer (Thermo Fisher Scientific, Waltham, MA, USA) and stored in aliquots at − 20 °C.

### Identification and analysis of tandem repeats

The Tandem repeats finder (TRF) program [18] was used with the amino acid sequence of the S3P strain to identify repetitive sequences present in *BbiTRAP*-*1*-*3*. A subset of sequences was manually selected from the TRF output of *BbiTRAP*-*1* according to the period size of the repeat (> 176 bp) and copy number (> 3). These repeats were further analyzed and documented.

Primers corresponding to the conserved 5′ and 3′ regions flanking approximately 20–30 bp from the *BbiTRAP*-*1* repeat region were manually designed using the S3P sequence. Primers (5′–3′) were: RepTRAPfw GACTCATCACAGAAAGCGCG and RepTRAPrv TCTTCCCTGCCTAGGTCTGA. These primers flank a region of repeats from positions 1392 to 2326 of the *BbiTRAP*-*1* nucleotide sequence. PCR amplifications were carried out using the genomic DNA from the *B. bigemina* strains mentioned above. DNA from the *B. bovis* R1A strain was used as a template negative control. Amplifications were performed using the T-Plus DNA Polymerase kit (INBIO HIGHWAY SA, Tandil, Argentina) containing 0.4 µmol of each primer, 0.2 mM of each deoxyribonucleotide triphosphate (Promega, Madison, WI, USA), 0.5 μg/μL bovine serum albumin, and 100 ng of genomic DNA. PCR analyses were performed in a 50-µl reaction mixture in a Bio-Rad MyCycler Thermal Cycler (BioRad, Hercules, CA, USA). The cycling conditions were denaturation at 94 °C for 3 min; annealing involving a touchdown step of 9 cycles at 95 °C for 1 min, 65 °C for 1 min, and 72 °C for 1 min and 10 s, with the annealing temperatures decreasing by 1 °C every cycle; followed by 27 cycles at 95 °C for 1 min, 59 °C for 1 min, and at 72 °C for 1 min and 10 s, and a final extension at 72 °C for 10 min.

Aliquots of 50 μl of each amplified product were electrophoresed in a 1% agarose gel, and the resulting DNA bands were stained with ethidium bromide. DNA bands were visualized on a UV image analyzer (BioRad) and documented. Bands of approximately 1028 and 800 bp were excised, purified, and cloned into the pGEM-T easy vector (Promega) according to standard protocols. The corresponding insert from purified plasmids was sequenced in both strands using vector primers. Sequences were deposited in GenBank under accession numbers MN450376–MN450383.

### Expression and purification of the recombinant BbiTRAP-1

For ease of expression, predicted transmembrane regions of high hydrophobicity of BbiTRAP-1 were identified using the TMHMM Server v.2.0 (www.cbs.dtu.dk/services/TMHMM) and removed from the final sequence. Therefore, a truncated DNA fragment of 1875 bp coding for 625 amino acids (corresponding to amino acids 320–944) was commercially synthesized (GenScript Biotech Corp., Piscataway, NJ, USA) and cloned between the* Eco*RI and* Bam*HI sites in the pET-28a vector (Sigma-Aldrich, St. Louis, MO, USA) along with an in-frame hexa histidine (6×His) tag for further protein purification. The plasmid map can be accessed in https://www.addgene.org/vector-database/2565/.

The *BbiTRAP*-*1*-pET28a construct was used to transform chemically competent *Escherichia* *coli* Rosetta (DE3) BL21 strain (Invitrogen, Carlsbad, CA, USA). Maximal expression of the protein was obtained after induction with 1 mM isopropyl β-D-1-thiogalactopyranoside (IPTG) at 20 °C overnight. Protein purification was performed using the ProBond™ Purification System according to the manufacturer’s instructions (Life Technologies, Carlsbad, CA, USA) to purify polyhistidine-containing recombinant proteins. The purified BbiTRAP-1 protein was analyzed by sodium dodecyl sulfate–polyacrylamide gel electrophoresis (SDS-PAGE) and the protein identity was confirmed by Western blot using a commercial anti-His tag antibody (Abcam, Cambridge, UK). The protein concentration was measured using the Pierce BCA Protein Assay kit (Thermo Fisher Scientific).

### Production of anti-BbiTRAP-1 polyclonal sera

Purified BbiTRAP-1 was used to immunize mice and bovines. In all cases, a pre-immunization bleeding was performed. Five male 7-week-old BALB/c mice were used and housed in cages with food and water provided ad libitum. Two 3-year-old female Aberdeen Angus bovines were housed in the plot field facilities at the Instituto Nacional de Tecnología Agropecuaria (INTA), Castelar, Argentina (34° 35′ 48.8″ S 58° 41′ 01.5″ W) and allowed to adapt to the conditions and handling for 1 week before starting the experiment. Trained and experienced animal technical assistants and care workers were in charge of the bovines. In mice, 30 µg of the antigen combined with complete Freund’s adjuvant (Sigma-Aldrich) were injected subcutaneously. Immunizations were repeated on days 14 and 21 with 30 µg of antigen combined with incomplete Freund’s adjuvant (Sigma-Aldrich). Blood samples were collected by submandibular bleeding at day 28, and sera was stored in aliquots at − 20 °C until use. All mice were anesthetized with isoflurane (Piramal Pharma Solutions, Lexington, KY, USA) and then killed by cervical dislocation.

For bovines, 100 μg of recombinant protein plus Montanide ISA-61VG adjuvant (Seppic, Paris, France) were injected 4 times every 21 days by the same route. Blood samples were collected by jugular venipuncture in Vacutainer™ tubes (Becton–Dickinson, Franklin Lakes, NJ, USA) at the end of the experiments, and sera were stored in aliquots at − 20 °C until use.

All the experiments were carried out under guidelines of the Institutional Committee for the Use and Care of Experimentation Animals (CICUAE–INTA protocols No. 17/2018 and 47/2018).

## BbiTRAP-1 protein expression analysis

The expression of TRAP-1 in *B. bigemina* merozoites was confirmed by Western blot analysis. Parasites were cultured at 37 °C in 5% CO_2_ in air in 25-cm^2^ flasks (JETBIOFIL, Guangzhou, China) containing a 5% (vol/vol) concentration of bovine erythrocytes from a healthy donor in medium 199 (GIBCO, Grand Island, NY, USA) supplemented with 40% autologous bovine serum. For the Western blot experiments, 50 ml of this *B. bigemina* culture was centrifuged at 2500*g* at 4 °C for 15 min followed by two washes with cold PBS (same centrifugation speed and temperature). The pellet was resuspended at 10% packed cell volume in PBS followed by addition of 1.13 ml of ice-cold distilled water per gram of wet pellet under continuous mixing by gentle shaking. The suspension was left for 1 min on ice, and then 0.11 volumes of 10× PBS were added to bring it back to the isotonic condition. The suspension was finally spun down, and the resulting pellet was washed twice with PBS, then resuspended in cracking buffer 2× and used for SDS-PAGE. After SDS-PAGE the gel was transferred onto nitrocellulose membranes (Merck & Co., Kenilworth, NJ, USA) using a wet blotting system, and the membrane was further blocked with 5% (w/v) skimmed milk in PBS for at least 2 h at room temperature or at 4 °C overnight with continuous shaking. The source of the primary antibodies was murine or bovine polyclonal sera, respectively. The secondary antibodies used were anti-murine or anti-bovine antibodies (goat anti-mouse IgG H&L [horseradish peroxidase, HRP]; Abcam) or rabbit anti-bovine IgG whole molecule (HRP) (Sigma-Aldrich) following standard protocols. After the membranes had been incubated for about 1 h at room temperature and then washed, the bands were developed using a chemiluminescent assy (enhanced chemiluminescence; Pierce™ ECL Western Blotting Substrate; Thermo Fisher Scientific).

### BbiTRAP-1 enzyme-linked immunosorbent assay

An indirect enzyme-linked immunosorbent assay (ELISA) using recombinant BbiTRAP-1 was performed following previously developed protocols [19]. Different concentrations of antigen (2, 5, 10, 20, 40, 80, and 100 ng/well) and serum dilutions had been checked in triplicate in previously run assays to optimize the assay conditions (data not shown). The optimal concentration of bound antigen was set at 40 ng/well, and serum dilutions were 1:1600 for mice and 1:10 for bovines.

Bovine sera from different geographic locations with a reported presence of ticks (Misiones, *n  *= 42, and Santa Fe, *n* = 27) were used. All sera had been previously tested as being positive for *B. bigemina* infection by an ELISA based on a merozoite lysate [20]. Sera from the tick-free area of the Buenos Aires region (*n* = 10) were included as negative controls; all of these sera had tested negative for *B. bovis and B. bigemina* antibodies by the merozoite-based ELISA. For specificity testing, 5 sera from *B. bovis*-positive animals were used. A threshold optical density (OD) value was calculated to discriminate *B. bigemina*-positive sera from *B. bigemina*-negative sera; this value was obtained by calculating the average OD of negative sera of triplicate data sets + 2 standard deviation values.

### In vitro neutralization assay

Inhibition of *B. bigemina* merozoite invasion of erythrocytes was performed as previously described for *B. bovis* [21]. Briefly, the *B. bigemina* PR strain (provided by the Animal Disease Research Unit of the U.S. Department of Agriculture laboratory at Washington State University, WA, USA) was cultured in a 96-well plate with 5% hematocrit. To that end, infected erythrocytes with 1% parasitemia were incubated with the sera to be analyzed in a culture medium containing 60% HL-1 medium (pH 7.2) and 40% of the aforementioned sera that had been previously heat-inactivated for 30 min at 56 °C. The culture was maintained at 37 °C in a 5% CO_2_ atmosphere for 72 h with changes of media every 24 h. Pre-immune mice and bovine sera were used as negative controls. Serum from a steer inoculated with the *B. bigemina* PR strain was used as the positive control. At the end of the incubation, the percentage of parasitized erythrocytes (PPE) was determined by flow cytometry using a Millipore Guava easyCyte HT flow cytometer (Merck Millipore, Burlington, MA, USA) and Flowing software, version 2.5 (Turku Bioscience Centre, Turku, Finland). Prior to sorting, samples were stained with hydroethidine (Dihydroethidium; Invitrogen) to stain the parasite’s DNA, following the manufacturers’ instructions. For the fluorescence-activated cell sorting (FACS), cell suspensions were diluted in PBS containing 0.2% sodium azide to obtain a flow rate of 2000–5000 cells/s, and each cell suspension was sampled in triplicate for statistical analysis. Measures of hydroethidine (blue fluorescence) were registered for all analyzed samples. The number of gated events was recorded on a fluorescence (BluFL2) versus side scatter (SSC) dot plot, where thresholds were set up based on the infected and non-infected controls [22]. The percentages of parasitemia inhibition (% pi) for the anti-BbiTRAP-1 antibodies were calculated with the following formula: % pi = 100 − ([PPE post-immunization serum/PPE pre-immunization serum] × 100). *P* values < 0.05 according to an independent Student’s *t* test were considered to be significant. The experiment was repeated twice.

### Indirect fluorescent antibody test

The indirect fluorescent antibody test (IFAT) was carried out essentially as described in the OIE (World Organization for Animal Health) manual [23], with minor modifications. Smears of infected red blood cells (iRBCs) from cultured *B. bigemina* parasites were fixed in ice-cold acetone for 30 s and air-dried. Pooled samples of polyclonal bovine sera anti-BbiTRAP-1 were diluted 1:100 in PBS; pooled samples of pre-immune bovine sera at the same dilution were used as negative controls.

All sera were incubated for 30 min in a humid chamber at 37 °C. After three washes (10 min in PBS and 5 min in bi-distilled water), fluorescein isothiocyanate (FITC)-conjugated anti-bovine IgG (H + L) (Sigma-Aldrich), diluted 1:100 in PBS containing Blue Evans (1:1000) and TO-PRO-3 (1:150) stains), was applied as a secondary antibody and incubated for 30 min in a humid chamber at 37 °C. The slides were washed twice for 10 min with PBS–Tween 20 0.05% and once for 5 min with bi-distilled water and then mounted with a coverslip in 1:2 glycerol–PBS. Epifluorescence was examined under a confocal microscope (Leica TCS SP5; Leica Microsystems, Mannheim, Germany).

## Results

### Identification and genetic characterization of the *TRAP* family in *Babesia* and *Theileria*

**Fig. 1:**
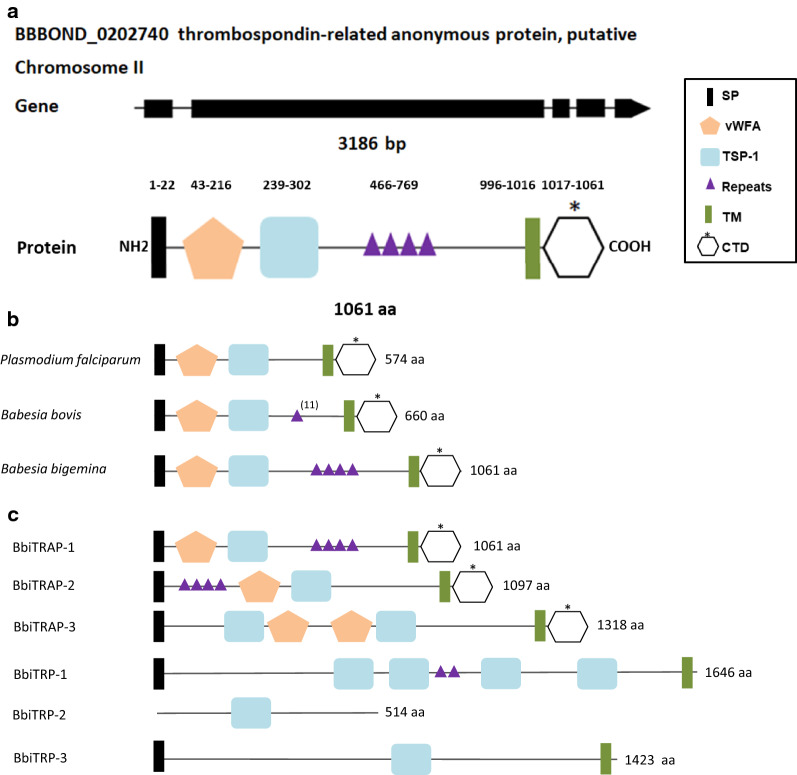
Sequence analysis of *Babesia bigemina TRAP*-*1* (*BbiTRAP*-*1*) gene. **a** Schematic representation of the *BbiTRAP*-*1* gene in the BOND strain with its five exons and the encoded polypeptide containing a signal peptide (*SP*), von Willebrand factor A (*vWFA*), a thrombospondin type-1 repeat domain (*TSP-1*), tandem repeats and the transmembrane region (*TM*) as well as a cytoplasmic tail domain (*CTD*). Asterisk indicates the presence of a tryptophan residue close to the CTD. **b** Schematic representation (not to scale) of the domains and repeats of three apicomplexan thrombospondin-related anonymous protein (TRAP) family proteins. Only long repeats (> 7 amino acids [*aa*]) are displayed as purple triangles. The triangles reflect the number of tandem repeat units with the exception of *Babesia bovis* TRAP-1 which has 11 repeat units in the T2Bo strain. The GenBank accession numbers of the corresponding genes are PF13_0201 (*Plasmodium falciparum*) and EDO06220.1 (*B. bovis*). The protein sizes in amino acids are shown on the right side.** c** Schematic representation (not to scale) of the domains and repeats of *B. bigemina* TRAP and thrombospondin-related protein (*TRP*) family proteins. Only long repeats (> 7aa) are displayed as purple triangles

**Fig. 2:**
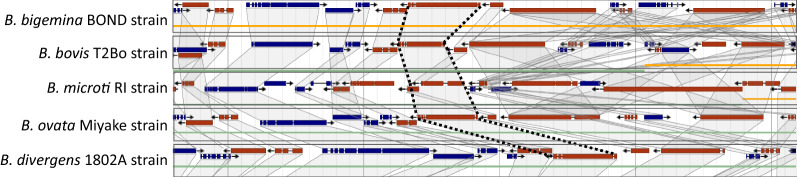
Synteny map analysis for *BbiTRAP*-*1*. Dotted lines indicate the location of *TRAP*-*1* orthologs in other *Babesia* species. Conservation of surrounding genes is shown as shaded areas

To identify the *BbiTRAP*-*1* coding sequence, we performed a TBLASTN search in the genome of *B. bigemina* (BOND strain) using the predicted amino acid sequence of the *B. bovis TRAP*-*1* gene as a query. This search resulted in the identification of an orthologous *TRAP*-*1* gene of 3186 bp that encodes a 1061-amino acid (aa) protein (BBBOND_0202740). This gene has five exons and is located in chromosome II, as in *B. bovis* (Fig. 1a). At the genome level, *BbiTRAP*-*1* as well as downstream and upstream surrounding genes are syntenic in comparison with *B. bovis*,* B. divergens*,* B. microti*, and *B. ovata* (Fig. 2).

The BbiTRAP-1 protein has a signal peptide at the N-terminus, the adhesive domains vWFA (approx. 200 residues), and TSP-1 (approx. 60 residues and also known as TRS) and the characteristic transmembrane domain (Fig. 1a). A metal ion-dependent adhesion site (MIDAS) motif is also present in the vWFA domain. This motif in BbiTRAP-1 is identical to the reported consensus sequence [7] and is composed of five non-contiguous amino acids, namely, Asp-Xaa-Ser-Xaa-Ser (where ‘Xaa’ can be any amino acid), that are brought together to accommodate a divalent cation. Even though the amino acid sequence of the TRAP-1 orthologs in *B. bovis* and *B. bigemina* share an amino acid identity of only 49 (62%), the modular composition and domain order in both species is the same as in its more distant ortholog, *P. falciparum* (Fig. 1b).

Previous studies in *Plasmodium* sp. show that TRAP proteins belong to a family with at least six members [24]. In *Apicomplexa* the genes encoding for these proteins are expanded in a lineage-specific fashion; therefore, we searched for the presence of other members of the *TRAP* gene family in the *Babesia* and *Theileria* genera. We first searched on PiroplasmaDB for all genes that coded for proteins containing the TSP-1 or vWFA domain since these functional domains are present in all TRAP proteins characterized to date [7]. Our criteria to assign a protein to the TRAP family included the presence of an acidic CTD with a conserved tryptophan residue near the C-terminal end of the protein, which is another typical feature of canonical apicomplexan TRAP proteins [14].

The results of this search, based on available current information, are shown in Additional file 1: Table T1. All species of *Babesia* and *Theileria* contain at least two TRAP genes in their genome. In general, the percentages of amino acid identity of TRAP proteins were found to be between 18.43 and 66.25% for TRAP-1 in *Babesia* spp. and between 42.66 and 82.91% for TRAP-1 in *Theileria* spp. *B. bovis* has the largest number of *TRAP* genes, with four members, and *B. canis* has only one *TRAP*-*2* gene. In particular, in *B. bigemina*, TRAP-2 has the same domain architecture as TRAP-1, whereas TRAP-3 has two TSP-1 domains and vWA domains, respectively (Fig. 1c).

A second search focused on identifying encoded proteins containing just the TSP-1 domain led us to also identify three new members of another group of TRAP-related proteins (Fig. 1c and Additional file 1: Table T2). These were named TRP after the report of these orthologous proteins in *Plasmodium berghei* [14]. Unlike TRAP, these TRP proteins lack the vWFA domain but contain a variable number of TSP-1 domains and a non-acidic CTD from which the conserved tryptophan residue is absent.

### Phylogenetic analysis

**Fig. 3:**
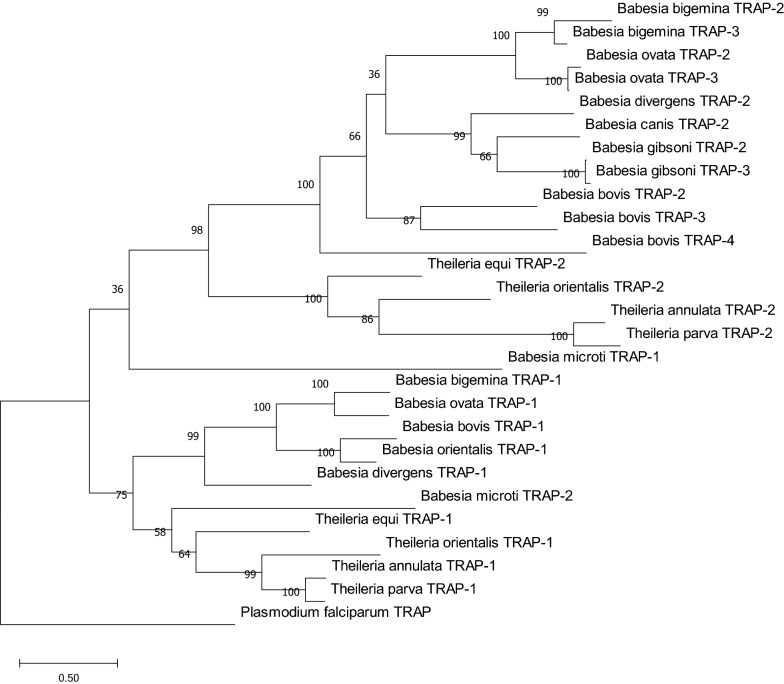
Phylogenetic tree constructed with the complete amino acid sequences of TRAP family members in *B. bigemina* together with paralogs and orthologs of closely related species. *Plasmodium falciparum* thrombospondin-related anonymous protein amino acid sequence was used as an outgroup. The tree was inferred using the maximum likelihood method. Bootstrap values after 1000 pseudo replicates are shown at each branch point

Phylogenetic analysis of sequences of the TRAP family in *B. bigemina* with paralogs and orthologs of available related species was performed using maximum likelihood estimation (Fig. 3), resulting in neighbor joining analysis retrieving a tree with similar topology (data not shown). This analysis, based on TRAP-1, showed that *B. ovata* is the species with the closest genetic relationship with *B. bigemina.* Both parasites are classified as a large-type *Babesia* that infect cattle. A further subclade including *B. bovis* and *B. orientalis* is also recognized with high bootstrap value (100%). In general, all TRAP-1 orthologs cluster together, with the exception of *B. microti*, which is considered as a sensu lato *Babesia* sp. In contrast, TRAP-2 and TRAP-3 protein sequences are grouped with their respective paralogs.

### Structural analysis of TRAP family

**Fig. 4:**
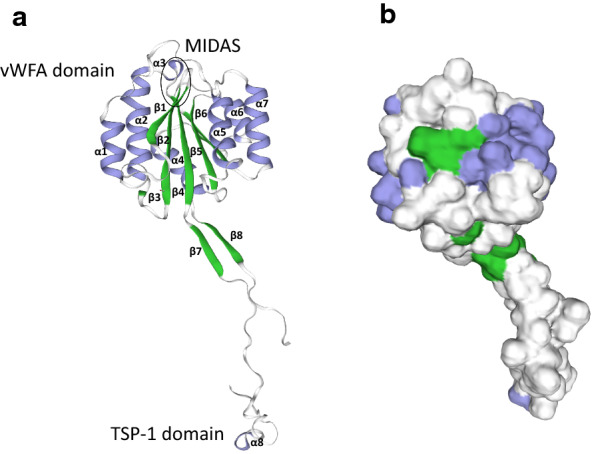
Hypothetical three-dimensional structure of BbiTRAP-1. **a** Cartoon structure of BbiTRAP-1 (residues 43–302). The α-helices are shown in violet and β-strands are indicated in green. The metal ion-dependent adhesion site (*MIDAS*) site for binding ligand is shown in a black circle. **b** BbiTRAP-1 surface structure

To further confirm the conservation of BbiTRAP-1-3 proteins with the already reported orthologs, the 3D structure of the three proteins was predicted in silico (Additional file 1: Fig. S1). Homology modeling was performed on the structure of a fragment of *Plasmodium vivax* sporozoite surface protein 2 (PDB code: 4hql.2.A) (also referred to as TRAP) [25], which was the candidate with the highest GMQE scores (0.60, 0.49, and 0.5 for TRAP-1, TRAP-2, and TRAP-3, respectively). The predicted 3D structures of the canonical vWFA and TSP-1 domains contain eight α-helices (α1–α8), eight β-strands (β1–β8), and the MIDAS site (Fig. 4). While the degree of amino acid conservation between BbiTRAP-1, 2, and 3 and sporozoite surface protein 2 is low (sequence similarity: 0.34, 0.30, and 0.30 for TRAP-1, TRAP-2, and TRAP-3, respectively), their overall predicted domain structures are well conserved.

### Sequence analysis of *BiTRAP*-*1* repeats

The TRF software was used to screen the *BbiTRAP*-*1* gene for the presence of tandem repeats based on previous findings of these repeats in the central part of the *TRAP*-*1* gene in *B. bovis* [18]. The BbiTRAP-1 protein contains four blocks of long amino acid repeats in the central portion of the protein. Two of these blocks have complete repeat modules of 84 aa each, while the last two are truncated (comprised of 60 and 73 aa, respectively).

**Fig. 5:**
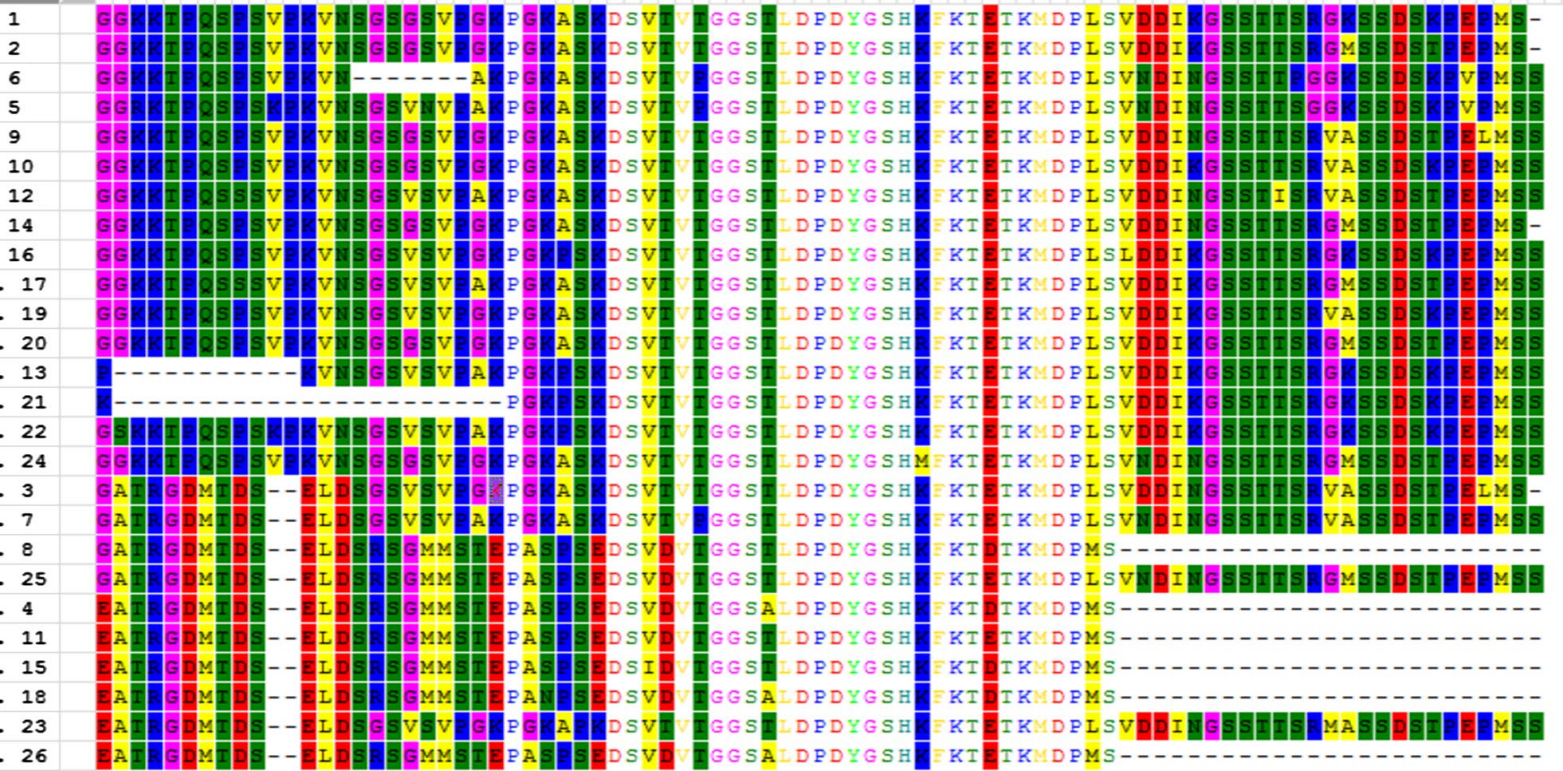
Amino acid alignment of the different repeat modules found in *BbiTRAP*-*1*. Each number on the left corresponds to a different type of repeat

 The tandem repeat motifs in TRAP-1 proteins were also analyzed in different *B. bigemina* strains obtained either from translated genomic data or from sequenced PCR products. Comparative analysis among strains from Argentina, Australia, Puerto Rico, Brazil, and Mexico determined that the tandem repeat modules varied in number and sequence among distinct isolates (Table 1). In order to facilitate the analysis of the variation of the repeat modules, a code number was assigned to each repeat. An alignment of all the repeats identified in this work is shown in Fig. 5.

**Table 1:** Variability of the TRAP-1 protein sequence of different *Babesia bigemina* strains

Strain	R I	R II	R III	R IV	Origin	Source
S3P	1	2	3	4	Argentina	Genome database [12]
S2A	13	14	3	15	Argentina	Own results
S1A	21	14	3	15	Argentina	Own results
M1A	22	14	3	15	Argentina	Own results
38	16	17	3	4	Argentina	Own results
S2P	16	12	23	11	Argentina	Own results
Brazil	1	14	3	18	Brazil	Own results
JG29	10	2	–	11	Mexico	Genome database [12]
Mexico_seed	19	20	–	11	Mexico	Own results
Nayarit	24	–	25	26	Mexico	Own results
PR	9	–	–	4	Puerto Rico	Genome database [12]
BOND	5	6	7	8	Australia	Genome database [12]

A number R I-R IV was assigned to each of the four repeat blocks shown in Fig. 1b and c for the BOND strain

The number and sequence of the repeats among strains is rather variable. Strains from Puerto Rico and Mexico have a shorter repeat region with only two and three repeat blocks, respectively. Some of the repeat modules are present in strains from a specific region, such as repeats 1, 3, 14 and 15 that were only found in Argentina and Brazil. Remarkably, all Argentinean attenuated strains analyzed here (S1A, S2A and M1A) show a relatively conserved repeat pattern where the first repeat module is variable but the last three ones (repeats 14, 3, 15) are exactly the same. All the repeats present in the Australian BOND and the Mexican Nayarit strains are unique and are not present in any other strain analyzed here.

Regarding BbiTRAP-2 and 3, four blocks of tandem repeats were found in BiTRAP-2, between amino acids 71 and 178. Two of these blocks have 27 amino acids each, while the last two have 25 and 28 amino acids, respectively. No repeats were identified in BiTRAP-3.

### Expression of recombinant BbiTRAP-1

**Fig. 6:**
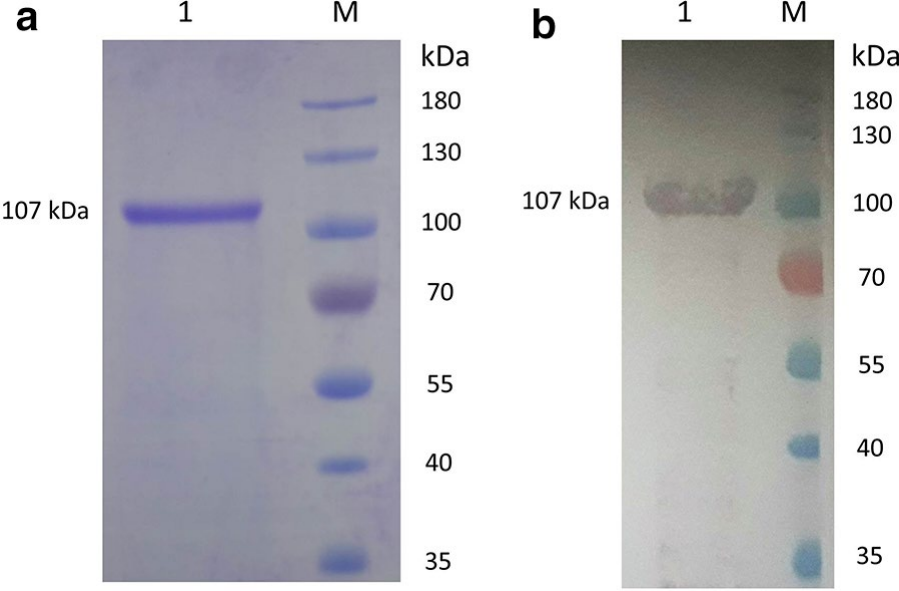
Expression of recombinant BbiTRAP-1 as a polyhistidine tag (His-Tag) protein. The purified protein fraction is shown following 12% sodium dodecyl sulfate-polyacrylamide gel electrophoresis.** a** Coomasie blue,** b** Western blot.* M* Molecular weight marker

For further characterization of TRAP-1 in *B. bigemina*, we generated a recombinant form of this protein containing only predicted extracellular regions and a His tag in order to facilitate expression and purification. The truncated recombinant BbiTRAP-1 protein (rBbiTRAP-1) was obtained at high yield and it could be purified under native conditions. After SDS-PAGE analysis (Fig. 6a, lane 1), a unique band of ~ 107 kDa, was observed. Even though the recombinant BbiTRAP-1 run with a higher than predicted molecular weight, positive Western blot results with the anti-His antibody confirmed the identity of the purified protein (Fig. 6b, lane 1).

### Evaluation of antibody reactivity against recombinant and native BbiTRAP-1

#### BbiTRAP-1 is recognized by antibodies from naturally infected cattle

**Fig. 7:**
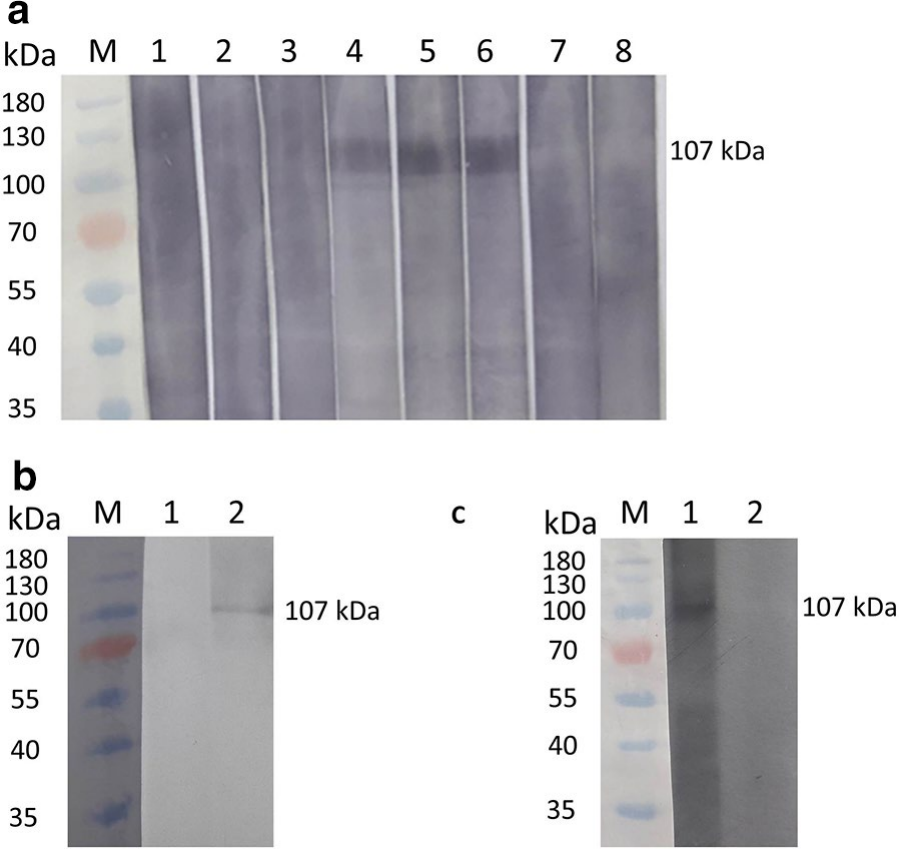
Evaluation of serum reactivity against recombinant (**a**) or native (**b**, **c**) BbiTRAP-1 by Western blot. **a*** Lanes*:* M* Molecular weight marker (kDa),* 1*,* 2*,* 3* sera from 3 different bovines infected with *B. bovis*,* 4*,* 5*,* 6* sera from 3 different bovines experimentally infected with *B. bigemina*,* 7*,* 8* sera from 2 bovines from a tick-free region (negative control). **b**.* Lanes*:* 1* Pre-immune mouse serum,* 2* anti-BbiTRAP-1 mouse serum. **c*** Lanes*:* 1* Anti-BbiTRAP-1 bovine serum,* 2* pre-immune bovine serum

In order to determine if antibodies present in the serum of *B. bigemina*-infected cattle would react with rBbiTRAP-1, we performed a Western blot analysis. Sera from these bovines specifically recognized the same band of  approximately 107 kDa protein that was obtained with the anti-His antibody (Fig. 7a, lanes 4–6). No reaction was observed with sera from uninfected cattle (Fig. 7a, lanes 7, 8) or from a bovine infected with *B. bovis* (Fig. 7a, lanes 1–3).

To further characterize the expression of the BbiTRAP-1 protein in merozoites, we initially tested the ability of sera from mice and bovines immunized with recombinant BbiTRAP-1 to recognize the native protein in immunoblot assays. In both animal models, antisera reacted with a single band of approximately 107 kDa in parasite lysates derived from *B. bigemina*-infected erythrocytes (Fig. 7b, lane 2 for mice and Fig. 7c, lane 1 for bovine). Accordingly, mice or bovine pre-immune sera did not react with any *B. bigemina* antigen (Fig. 7b, lane 1 and Fig. 7c, lane 2). Taken together, the data confirm the expression of BbiTRAP-1 in blood stages of the parasite.

#### Truncated BbiTRAP-1 is not immunodominant in *B. bigemina*-infected bovines

An in house indirect ELISA was developed to further assess the immunogenicity of BbiTRAP-1 using sera from *B. bigemina*-infected animals. For this purpose, a set of 69 sera from *B. bigemina*-infected herds from two different geographic origins (Misiones and Santa Fe, Argentina) was used. The results showed that 29.62% of the sera samples from experimentally-infected bovines and 42.85% of those from naturally-infected bovines recognized BbiTRAP-1 in this ELISA (Additional file 1: Fig. S2). The specificity of the test was optimal (100%), and the sensitivity had the same values as above since no false negative results were obtained. This pattern of reactivity suggests that rBbiTRAP-1 is not an immunodominant antigen and would not be an optimal candidate for developing novel serological diagnostic assays.

#### BbiTRAP 1 contains neutralization-sensitive epitopes

To determine whether BbiTRAP-1 has neutralization-sensitive B-cell epitopes, we performed an in vitro invasion assay using anti-recombinant BbiTRAP-1 murine and bovine hyperimmune sera. After 72 h of the initiation of the cultures, antibodies against BbiTRAP-1 present in both sera had neutralized invasion by 39.94% in the mice sera (Additional file 1: Fig. S3) and by 46.92% in bovine sera.

We observed some unspecific effect of the mice pre-immune serum in the infection capacity of the parasites (Additional file 1: Fig.S3a [panel c], b). However, anti-BbiTRAP-1 antibodies (Additional file 1: Fig. S3a [panel d], b) do have a significant inhibitory effect on the rate of in vitro growth of the parasite when compared to both preimmune sera and control non-treated cultures (*p * < 0.05).

Confirmation of the presence of in vitro neutralization-sensitive epitopes in BbiTRAP-1 suggests that this protein may also be exposed to neutralizing antibodies during infection and that these could be targeted as a possible component of a subunit vaccine against *B. bigemina*.

#### BbiTRAP-1 is expressed in intraerythrocytic merozoites

**Fig. 8:**
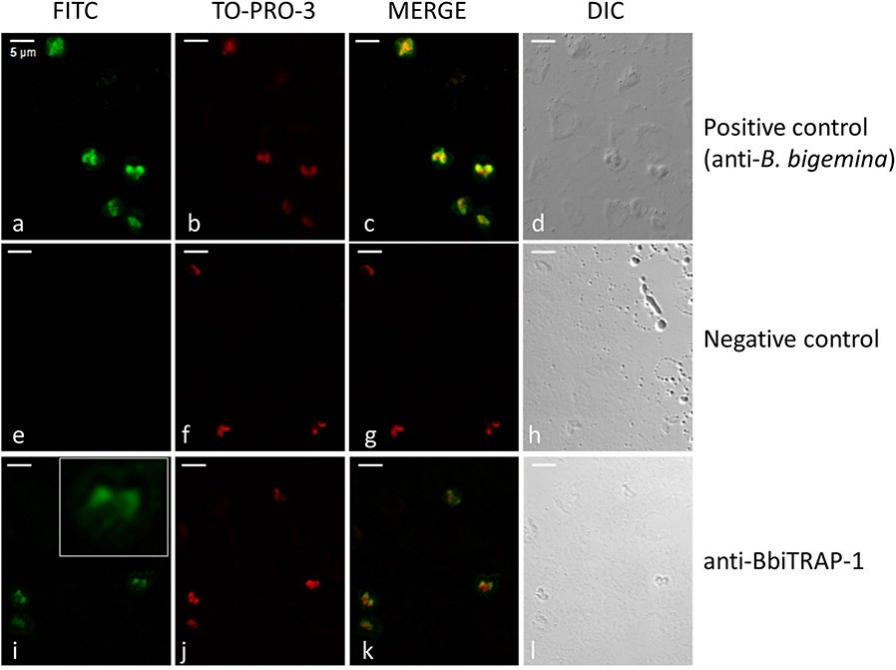
Indirect immunofluorescence assay by confocal laser microscopy of *B. bigemina* with antibodies against recombinant BbiTRAP-1. Smears of *B. bigemina*-infected merozoites were incubated with sera from a bovine experimentally infected with *B. bigemina* (**a–d**), a control bovine immunized with adjuvant alone (**e**–**h**), and a bovine immunized with recombinant BbiTRAP-1 (**i**–**l**). **c**, **g**, **k** Fluorescein isothiocyanate (*FITC*)-conjugated anti-bovine IgG and TO-PRO-3 DNA stain were used to create a merged image. Inset in** i** Enlargement of duplicated *B. bigemina* merozoites reacting with anti-BbiTRAP-1.* DIC* Differential interference contrast microscopy images are located on the far right and were used to confirm the location and number of individual intracellular parasites. Scale bars: 5 μm

We also analyzed the localization of BbiTRAP-1 in intracellular merozoites. The pattern of reactivity of bovine serum raised against recombinant BbiTRAP-1 was tested by IFAT using fixed smears of *B. bigemina*-infected erythrocytes. The green fluorescence of the FITC-anti-bovine conjugate localized with a strong focal signal in the wider region of the pear-shaped merozoites compared with the rest of the parasite’s body (Fig. 8i). This positive signal was not observed when iRBCs were incubated with a *B. bigemina*-negative bovine serum (Fig. 8e). Both anti-BbiTRAP-1 and anti-*B. bigemina* antisera showed a very similar staining pattern with the fluorescence appearing to be concentrated at the bottom side of the parasites.

## Discussion

BbiTRAP‐1 is a novel, well-conserved antigen of *Babesia bigemina* with structural and antigenic characteristics similar to those of several adhesin orthologs already described. These include proteins of diverse origin, such as the *Plasmodium* TRAP and the circumsporozoite protein as well as *T. gondii* micronemal proteins [7]. We propose that both TRAP and TSP are members of a TRAP superfamily and share a common ancestral origin and non-overlapping functions. The widespread conservation of this multigene TRAP superfamily, including canonical TRAP and the TRP families decribed herein, among distinct apicomplexan parasites underscore an important functional role and relevance for parasite survival. Whether TRP proteins are functional equivalents of TRAP, or if they play different roles remains unknown.

Indeed, proteins containing adhesive domains like TRAPs allow the parasite to interact with receptors present at the host cell surface and are linked to the glideosome, a molecular machine necessary for parasite motility and host-cell invasion [5]. The importance of adhesive domains was demonstrated in recent studies on *Plasmodium berghei* TRAP in which deletion of the vWFA domain abolished gliding motility, mosquito salivary gland invasion, and subsequent mouse infection [26]. For these reasons, these proteins could be important targets for immunological or chemotherapeutic interventions.

Our genomic analysis showed that the *BbiTRAP*-*1* gene is in fact part of a family composed of two additional members, designated as *BbiTRAP*-*2* and *BbiTRAP*-*3*. According to what is widely known in *Apicomplexa*, we confirm here the observation that the TRAP family in Piroplasmida possesses two or three genes depending on the genus. Whether the expression of TRAP family proteins is stage-specific, as is reported in *Plasmodium*, will demand further studies.

Expression of TRAP-2 in merozoites has been reported previously, but only for *B. bovis* and *B. orientalis* [9, 27]; to our knowledge, no other study has described the presence of these proteins in other members of Piroplasmida. In this context, our data provide additional information on this gene family for future characterization studies not only in *Babesia* but in *Theileria* sp. as well. The data described here extend also to the new *TRP* gene family identified by us in both genera through a domain-driven bioinformatic search. In relation to TRP function, it has been demonstrated that the *P. berghei* TRP ortholog is involved in oocyst egress and salivary gland invasion [14]; therefore, further studies in tick stages will shed light on the functional relevance of these proteins in Piroplasmida.

Regarding *BbiTRAP*-*1*, the chromosomal region that contains this gene is syntenic between *B. bigemina* and the other *Babesia* species analyzed. This observation indicates that this region of the chromosome is well conserved across the *Babesia* genus and that recombination would be infrequent for this region of the genome. At the gene level, the number of exons in *BbiTRAP*-*1* also coincides with their orthologs in *B. bovis*,* B. orientalis*, and *B. ovata*, suggesting a closer genetic relationship among these members of the genus. In this sense, the results of our phylogenetic analysis of *TRAP*-*1* in *Babesia* sp. support the hypothesis of a closer association among these species by showing that *B. bigemina*,* B. orientalis*,* B. bovis*, and *B. ovata* form a separate clade apart from *B. divergens.* It is interesting to note that the *TRAP*-*1* tree reflects mostly the taxonomic relationships of members of the order Piroplasmida based on studies of mitochondrial and 18S genes [28]. In both cases, *Babesia* species of the sensu stricto group form a separate phylogenetic clade apart from *Theileria* sp. and *B. microti*.

The presence of only one *TRAP*-*2* member in *B. canis* can be attributed to a sequencing artifact due to the fragmentation of the *B. canis* genome into a large number of scaffolds [29]. It has been reported that incorrect genome assemblies result in inferring a wrong number of genes belonging to a gene family [30], so the number of *TRAP* genes, transmembrane regions, and acidic CTD in *B. canis* should be further revised.

Even though the overall amino acid identity of the BbiTRAP-1 protein compared with that of other *Babesia* species is relatively low, the sequence conservation and tertiary structure is very high in key adhesive domains, such as vWFA and TSP-1. This conservation goes beyond the *Babesia* genus, as reflected by the good fitting of the predicted BbiTRAP-1 3D structure with the sporozoite surface protein 2 of *Plasmodium vivax*, confirming the functional importance of this protein in critical processes not only in the *Babesia* genus, but also among other apicomplexan parasites. Conserved domains like the vWFA, with similar overall structures in distant phyla, have been reported to be polyspecific in *Apicomplexa*, providing the parasites with the versatility to interact with multiple ligands and migrate into diverse organs [26].

Another conserved feature of BbiTRAP-1 and other adhesive proteins is the presence of tandem repeat units in the central part of the protein. The presence of repeats or of tandem duplication of adhesive domains has been associated with parasitic evolution strategies to form high avidity complexes for host cell invasion [31] or to be a smokescreen to escape from the vertebrate host immune system [32]. The presence of these repeats has already been described for *B. bovis TRAP*-*1* [33] where this high ratio of variation was exploited for use as a molecular marker. In our study the variation in sequence and order of the repeat units in *BbiTRAP*-*1* could also be applied for high-resolution molecular fingerprinting of *B. bigemina* isolates. Further analysis at the genomic level will determine if the high conservation of *BbiTRAP*-*1* repeat modules in vaccine strains of Argentina is a more widespread phenomenon, and if it could reflect a process of reduced genome diversity after attenuation, similar to what has been reported for *B. bovis* [34].

The truncated recombinant BbiTRAP-1 was found to have a higher than expected molecular size. This phenomenon has been reported for acidic proteins of eukaryotic origin and been attributed to changes in protein mobility due to low binding of SDS, producing insufficient denaturation and slower migration in the gel [35, 36]. Consistently, the recombinant BbiTRAP-1 obtained in this work was found to have 17.9% of acidic amino acids and a theoretical pI of 4.72.

Testing the reactivity of anti-rBbiTRAP-1 in Western blot against a *B. bigemina* lysate resulted in the recognition of native *B. bigemina* merozoite protein with the predicted size of BbiTRAP-1, suggesting that this protein is actually expressed in the blood stages of the parasite. This result is fully consistent with the identification of anti-rBbiTRAP-1 antibodies in infected cattle and the strong IFAT signal. Furthermore, the immunofluorescence assay using bovine antisera demonstrated that this protein is expressed in intra-erythrocytic merozoites, with a stronger signal in the region of the apical complex where adhesion proteins for initial attachment are frequently located. The presence of a signal peptide in BbiTRAP-1 is also compatible with its involvement in a possible secretion pathway via association with the apical complex organelles of the parasite.

One of the major drivers in *Babesia* studies is the searcg for immunodominant antigens that can be used in serological diagnostics. In Argentina, the only serological test used routinely for *B. bigemina* diagnosis is an indirect ELISA based on a merozoite lysate [20] that has two major limitations: (1) the need for parasite culture maintenance or infected bovines and (2) difficulty in standardization. The ELISA results with the truncated form of BbiTRAP-1 had values of sensitivity of 29.62–42.85%. These values are below those considered to be optimal for an ELISA test and indicate that at least the selected portion of BbiTRAP-1 expressed by us is not suitable for a diagnostic test and that the epitopes present in this protein are not immunodominant. If BbiTRAP-1 is in fact a subdominant antigen, it may be a molecule that is essential for parasite survival and, therefore, a promising candidate for vaccine development.

The involvement of members of the TRAP family during the erythrocyte invasion by merozoites has been previously demonstrated in *B. bovis* [6, 27] and in other *Babesia* species [8–10]. Our results showing an important reduction in parasite invasion assays with anti-TRAP-1 antibodies support the hypothesis of the presence of surface-exposed neutralizing B-cell epitopes, which is in accordance with these previously published reports. As the recombinant BbiTRAP-1 protein obtained in this work lacks hydrophobic portions in the N- and C- terminal regions, it can be assumed that these epitopes are located outside these regions. Further experiments involving epitope mapping will be required to determine the exact location of these epitopes.

In summary, we have characterized a novel TRAP-1 homolog expressed by merozoites of *B. bigemina* and provide evidence that this antigen has B-cell neutralizing epitopes that are exposed during infection and which could play a key role in parasite invasion. These functional characteristics together with its possible antigenic subdominance suggest that BbiTRAP-1 is a rational candidate for developing a subunit vaccine against *B. bigemina.*

## Conclusions

In this study we have identified the *BbiTRAP* and *BbiTRP* gene families in several *Babesia* and *Theileria* species and defined these genes as members of a gene superfamily. We have also characterized BbiTRAP-1 as a novel antigen of *B. bigemina* with structural and antigenic characteristics similar to those of its orthologs in other *Apicomplexa*. The presence of adhesive domains, perceived functional significance, and high level of conservation, together with the identification of surface-exposed neutralization-sensitive B-cell epitopes and its possible immunological subdominance, suggests that BbiTRAP-1 should be included as a subunit vaccine candidate against *B. bigemina*.

## Supplementary information

**Additional file 1: Tables T1** and **T2.** Summary of relevant information of all TRAP (Table T1) and TRP (Table T2) family members. Accession numbers are from PiroplasmaDB database except those marked with a superscript a (^a^) which are NCBI accession numbers. ^b^See reference of TRAP-2 of *B. orientalis* [9]. ^c^ID obtained from genome annotation. Domains are: SP, vWFA, TSP-1, TM, and acidic CTD with a tryptophan residue close to the C-terminus (*W*). Orthology group is according to OrthoMCL database release 6.1.* N/A* Not available. **Fig. S1** Hypothetical 3D structure of BbiTRAP-1-3 as cartoon and surface structures. **Fig. S2** Analysis of samples from *Babesia bigemina*-infected bovines by the merozoite and BbiTRAP-1 ELISA tests. **Fig. S3** **a** Flow cytometry dot plots associated to the neutralization assays. Hydroethidine-stained cultures were analyzed by flow cytometry and the number of gated events was recorded on a fluorescence (BluFL2) versus side scatter (SSC) dot plot. The assays were performed in triplicate and the experiment was repeated twice. To set up the thresholds of the gating strategy we used a non-infected control containing only RBC (*a*) and a control of iRBC without the addition of serum (*b*). Pre-immunization (*c*) and post-immunization serum (*d*) refer to the mice inoculation with BiTRAP-1.** b** Average parasitemias and standard deviations were calculated from triplicates, as described in** a**. Asterisks represent statistically significant differences according to Student’s* t* test (*P *< 0.05)

## Data Availability

The nucleotide sequences generated during this study were submitted to the GenBank database under the accession numbers MN450376–MN450383.

## Abbreviations

iRBC: Infected red blood cell
MIDAS: Metal ion-dependent adhesion site
PPE: Percentage of parasitized erythrocyte
TRAP-1: Thrombospondin-related anonymous protein type 1
TRP: Thrombospondin-related proteins
TSP-1: Thrombospondin type-1 repeat
vWFA: Von Willebrand factor A
