# Supplementary material for: Identification and characterization of a Babesia bigemina thrombospondin-related superfamily member, TRAP-1: a novel antigen containing neutralizing epitopes involved in merozoite invasion
Source: Parasit Vectors. 2020 Dec 1;13:602. doi: 10.1186/s13071-020-04469-5 (PMC7705850; doi:10.1186/s13071-020-04469-5)

Supplementary Tables T1 and T2: summarizes relevant information of all TRAP and TRP family members. Accession numbers are from PiroplasmaDB database except those with (a) which indicate NCBI accession numbers. b. See reference of TRAP-2 of B. orientalis (9). c. ID obtained from genome annotation. Domains: SP, vWFA, TSP-1, TM, acidic CTD with a tryptophan residue close to the C-terminus (W). Orthology group according to OrthoMCL database release 6.1. N/A: no available.

Supplementary Table T1

	TRAP Family	Accession numbers	SP	vWFA	TSP-1	TM	acidic CTD	W	Orthology group
B. bigemina	TRAP-1	BBBOND_0202740	Yes	1	1	Yes	Yes	Yes	OG6_115645
	TRAP-2	BBBOND_0202800	Yes	1	1	Yes	Yes	Yes	OG6_194574
	TRAP-3	BBBOND_0202760	Yes	2	2	Yes	Yes	Yes	OG6_194574
B. bovis	TRAP-1	BBOV_II002650	Yes	1	1	Yes	Yes	Yes	OG6_115645
	TRAP-2	BBOV_II002890	Yes	2	2	Yes	Yes	Yes	OG6_194574
	TRAP-3	BBOV_II002630	Yes	2	2	Yes	Yes	Yes	OG6_194574
	TRAP-4	BBOV_II002870	Yes	1	No	Yes	Yes	Yes	OG6_194574
B. canis	TRAP-2	Bc-CHIPZ-H002772[c]	Yes	1	1	No	No	No	OG6_194574
B. orientalis	TRAP-1	AYJ18799.1[a]	Yes	1	1	Yes	Yes	Yes	OG6_115645
	TRAP-2	See reference[b]	N/A	N/A	N/A	N/A	N/A	N/A	N/A
B. gibsoni	TRAP-2	BAI66064.1[a]	No	1	1	Yes	Yes	Yes	OG6_194574
	TRAP-3	BAB68553.2[a]	Yes	1	1	Yes	Yes	Yes	OG6_194574
B. divergens	TRAP-1	Bdiv_010490	Yes	1	1	Yes	Yes	Yes	OG6_115645
	TRAP-2	Bdiv_010470	Yes	1	1	Yes	Yes	Yes	OG6_194574
B. microti	TRAP-1	BmR1_04g08630	Yes	1	1	Yes	Yes	Yes	OG6_115645
	TRAP-2	BmR1_04g08640	No	1	3	Yes	Yes	Yes	OG6_106501
B. ovata	TRAP-1	BOVATA_031530	Yes	1	1	Yes	Yes	Yes	OG6_115645
	TRAP-2	BOVATA_031480	Yes	1	1	Yes	Yes	Yes	OG6_194574
	TRAP-3	BOVATA_031510	Yes	2	2	Yes	Yes	Yes	OG6_194574
T. annulata	TRAP-1	TA07755	No	1	1	Yes	Yes	Yes	OG6_109573
	TRAP-2	TA07750	Yes	1	1	Yes	Yes	Yes	OG6_129789
T. equi	TRAP-1	BEWA_005710	Yes	1	1	Yes	Yes	Yes	OG6_109573
	TRAP-2	BEWA_005690	Yes	1	No	Yes	Yes	Yes	OG6_129789
T. orientalis	TRAP-1	TOT_040000591	Yes	1	1	Yes	No	No	OG6_109573
	TRAP-2	TOT_040000593	No	1	1	No	No	Yes	OG6_109272
T. parva	TRAP-1	TP04_0306	No	1	1	Yes	No	No	OG6_109573
	TRAP-2	TP04_0305	Yes	1	No	Yes	Yes	Yes	OG6_129789

Supplementary Table T2

	TRP Family	Accession numbers	SP	vWFA	TSP-1	TM	Orthology group
B. bigemina	TRP-1	BBBOND_0103660	Yes	No	4	Yes	OG6_130928
	TRP-2	BBBOND_0105380	No	No	1	No	OG6_160841
	TRP-3	BBBOND_0103630	Yes	No	1	Yes	OG6_171449
B. bovis	TRP-1	BBOV_II006510	Yes	No	3	Yes	OG6_130928
	TRP-2	BBOV_IV008000	No	No	1	No	OG6_160841
	TRP-3	BBOV_II006540	No	No	2	Yes	OG6_171449
B. divergens	TRP-1	Bdiv_004450	Yes	No	3	Yes	OG6_130928
	TRP-2	Bdiv_020650	No	No	1	No	OG6_160841
	TRP-3	Bdiv_004480c	Yes	No	2	Yes	OG6_171449
B. microti	TRP-1	BMR1_03g00437	Yes	No	2	Yes	OG6_130928
	TRP-2	BmR1_04g09041	No	No	1	Yes	OG6_160841
	TRP-3	BMR1_03g00451	No	No	1	Yes	OG6_171449
B. ovata	TRP-1	BOVATA_022550	Yes	No	3	Yes	OG6_130928
	TRP-3	BOVATA_022570	Yes	No	3	Yes	OG6_171449
T. annulata	TRP-1	TA14200	Yes	No	1	Yes	OG6_130928
	TRP-2	TA16575	No	No	1	Yes	OG6_160841
	TRP-3	TA14215	Yes	No	1	Yes	OG6_171449
T. equi	TRP-1	BEWA_024230	Yes	No	3	Yes	OG6_130928
	TRP-3	BEWA_024280	No	No	2	No	OG6_171449
T. orientalis	TRP-1	TOT_020000606	Yes	No	1	No	OG6_130928
	TRP-2	TOT_010000937	No	No	1	Yes	OG6_160841
	TRP-3	TOT_020000608	No	No	2	Yes	OG6_171449
T. parva	TRP-1	TP02_0615	No	No	1	Yes	OG6_130928
	TRP-2	TP01_0984	No	No	1	No	OG6_160841
	TRP-3	TP02_0622	No	No	1	No	OG6_171449

Supplementary Figure S1: Hypothetical 3D structure of BbiTRAP-1-3 as cartoon and surface structures.

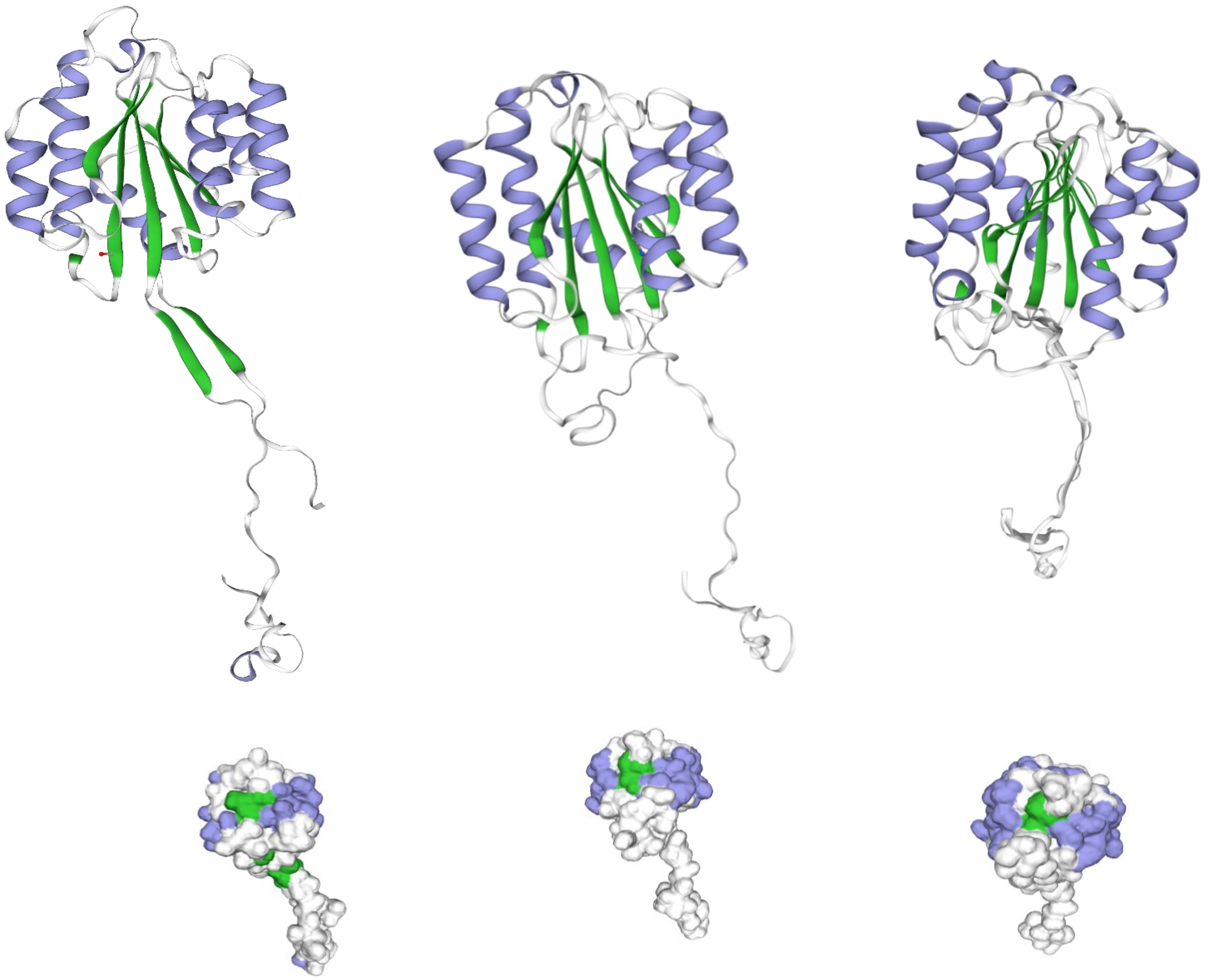

TRAP-1 TRAP-2 TRAP-3

Supplementary Figure S2: Analysis of samples from *Babesia bigemina* infected bovines by the merozoite and BbiTRAP-1 ELISA tests.

Experimentally-infected bovines		Merozoite-ELISA		
		+	-	Total
BiTRAP-1-ELISA	+	8	0	8
	-	19	10	29
	Total	27	10	37

Naturally-infected bovines		Merozoite-ELISA		
		+	-	Total
BiTRAP-1-ELISA	+	18	0	18
	-	24	10	34
	Total	42	10	52

Supplementary Figure S3: (A) Flow cytometry dot plots associated to the neutralization assays. Hydroethidine stained cultures were analyzed by flow cytometry and the number of gated events was recorded on a fluorescence (BluFL2) versus side scatter (SSC) dot plot. The assays were performed in triplicate and the experiment was repeated twice. To set up the thresholds of the gating strategy a non-infected control containing only RBC (panel a) and a control of iRBC without the addition of serum (panel b) were used. Pre-immunization (panel b) and post-immunization serum (panel d) refer to the mice inoculation with BiTRAP-1. (B) Average parasitemias and standard deviations were calculated from triplicates, as described in (A). Asterisks represent statistically significant differences according to Student´s t test (*p<0.05*).

A

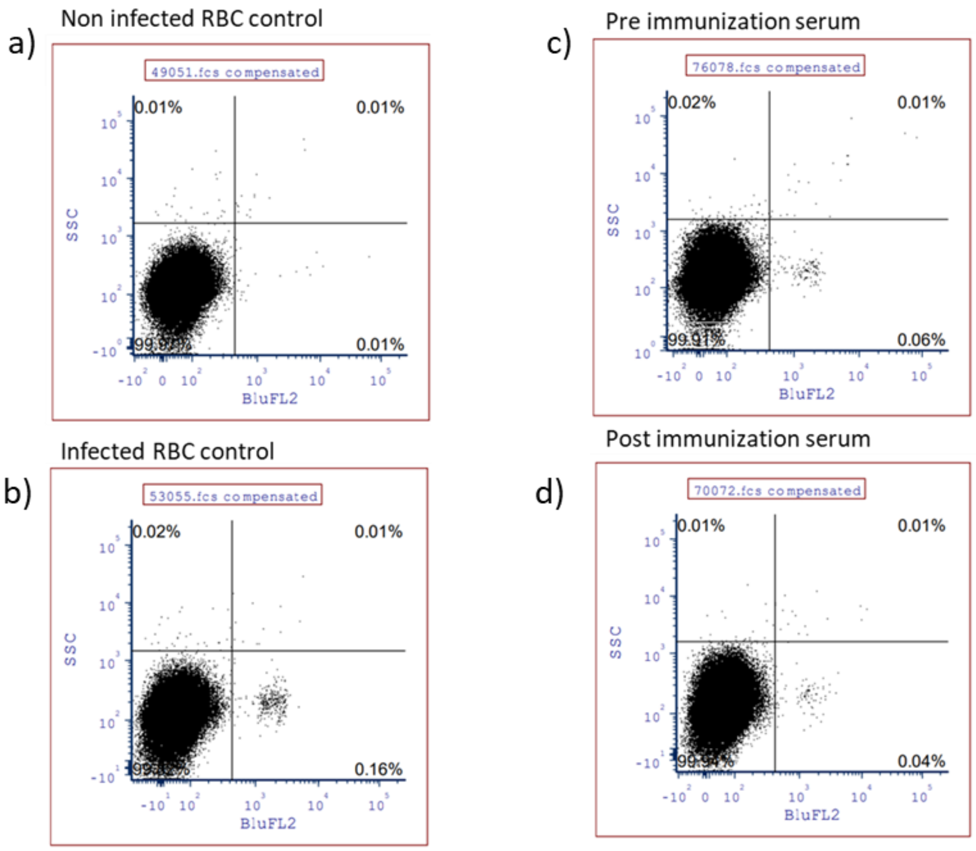

B

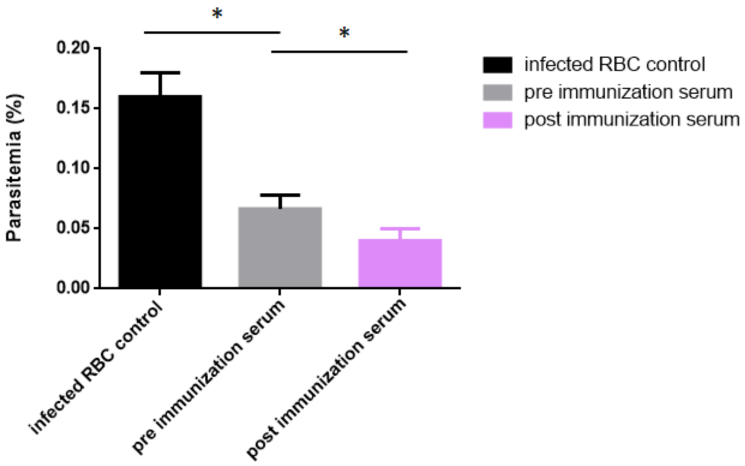

Supplement: Supplementary file 1 — Additional file 1: Tables T1 and T2. Summary of relevant information of all TRAP (Table T1) and TRP (Table T2) family members. Accession numbers are from PiroplasmaDB database except those marked with a superscript a (a) which are NCBI accession numbers. bSee reference of TRAP-2 of B. orientalis [9]. cID obtained from genome annotation. Domains are: SP, vWFA, TSP-1, TM, and acidic CTD with a tryptophan residue close to the C-terminus (W). Orthology group is according to OrthoMCL database release 6.1. N/A Not available. Fig. S1 Hypothetical 3D structure of BbiTRAP-1-3 as cartoon and surface structures. Fig. S2 Analysis of samples from Babesia bigemina-infected bovines by the merozoite and BbiTRAP-1 ELISA tests. Fig. S3 a Flow cytometry dot plots associated to the neutralization assays. Hydroethidine-stained cultures were analyzed by flow cytometry and the number of gated events was recorded on a fluorescence (BluFL2) versus side scatter (SSC) dot plot. The assays were performed in triplicate and the experiment was repeated twice. To set up the thresholds of the gating strategy we used a non-infected control containing only RBC (a) and a control of iRBC without the addition of serum (b). Pre-immunization (c) and post-immunization serum (d) refer to the mice inoculation with BiTRAP-1. b Average parasitemias and standard deviations were calculated from triplicates, as described in a. Asterisks represent statistically significant differences according to Student’s t test (P < 0.05) [file 13071_2020_4469_MOESM1_ESM.pdf]
